# Effect of *ESR1* and *ESR2* polymorphisms on ovarian response in assisted reproductive technology cycles: a systematic review and meta-analysis

**DOI:** 10.1038/s41598-026-53350-5

**Published:** 2026-06-05

**Authors:** Khan Zahara Fathima, Vani Lakshmi R., Stella Irungu, Aditi Gupta, Vijay Shree Dhyani, Prashanth Adiga, Srabani Mukherjee, Satish Kumar Adiga, Shubhashree Uppangala

**Affiliations:** 1 Division of Reproductive Genetics, Department of Reproductive Science, Kasturba Medical College, Manipal Academy of Higher Education, Manipal, Karnataka India; 2 Centre for Digital Health, Applied Research and Technology, Prasanna School of Public Health, Manipal Academy of Higher Education, Manipal, Karnataka 576104 India; 3 Department of Reproductive Health and Biology, Institute of Primate Research, Nairobi, Kenya; 4 Kasturba Medical College, Manipal Academy of Higher Education, Manipal, Karnataka India; 5 Department of Reproductive Medicine and Surgery, Kasturba Medical College, Manipal Academy of Higher Education, Manipal, Karnataka India; 6 Department of Molecular Endocrinology, National Institute for Research in Reproductive Health, Indian Council of Medical Research, Mumbai, Maharashtra India; 7 Centre of Excellence in Clinical Embryology, Department of Reproductive Science, Kasturba Medical College, Manipal Academy of Higher Education, Manipal, Karnataka India

**Keywords:** ART, Controlled ovarian stimulation, Estrogen receptor, Single nucleotide polymorphisms, Ovarian response, Diseases, Genetics, Medical research

## Abstract

Genetic factors contribute to poor ovarian response (POR) in assisted reproductive technology cycles, with estrogen receptor gene polymorphisms (*ESR1* and *ESR2*) being explored as a causative factor in many studies. However, there is no systematic review and meta-analysis conducted on these studies to assess the association of these receptor polymorphisms on POR. This systematic review and meta-analysis evaluated the association between *ESR1* and *ESR2* single nucleotide polymorphisms (SNPs) and ovarian response in women undergoing controlled ovarian stimulation (COS). The study was registered with PROSPERO and conducted according to PRISMA guidelines. 11 eligible studies were included. Among the various SNPs, meta-analysis focused on *ESR1* rs2234693 and rs9340799, and *ESR2* rs4986938. *ESR1* rs2234693 C‑allele carriers showed significantly increased odds of POR in both heterozygote (pooled log odds ratio (LOR) = 0.99, standard error (SE) = 0.213; odds ratio (OR) = 2.69, *p* < 0.001)) and dominant models (LOR = 1.69, SE = 0.449, OR = 5.41, *p* < 0.001), with consistent findings in European populations. Associations for *ESR1* rs9340799 and *ESR2* rs4986938 with POR showed substantial heterogeneity. Overall, *ESR1* rs2234693 may be associated with POR, in women undergoing COS. Further large-scale studies with appropriate study designs are warranted to clarify the potential role of *ESR* polymorphisms in COS outcomes.

**Supplementary Information:**

The online version contains supplementary material available at 10.1038/s41598-026-53350-5.

## Introduction

Poor ovarian response (POR) occurs when patients demonstrate a low or inadequate response to ovarian stimulation, resulting in a reduced number of retrieved oocytes and consequently lowering the likelihood of pregnancy^1^. The prevalence of POR varies widely, affecting approximately 9–24% of women undergoing assisted reproductive technology (ART). Some of the risk factors that are associated with POR are diminished ovarian reserve, advanced maternal age, and genetic predisposition^2,3^. Genetic differences observed as a consequence of single nucleotide polymorphisms (SNPs) in genes associated with female fertility have been associated with POR pathophysiology^4,5^. Studying such SNPs associated with POR will allow for screening, identification, and optimization of stimulation protocols specifically tailored for these patients^6,7^.

The key genes implicated in ovarian response during exogenous follicle stimulating hormone (FSH) stimulation are estrogen receptors (ESRs). This is mainly because estrogens expand the action of FSH on granulosa cells by supporting their proliferation and augmenting their expression of FSH receptors^8^. Furthermore, reports have suggested their key role in embryonic development, endometrial cell growth and differentiation, thereby regulating the implantation process and pregnancy^9–12^. The biological effects of estrogens are mediated through binding to specific nuclear receptor proteins known as ESRs, which function as ligand-activated transcription factors^13^. These receptors regulate gene expression by binding to estrogen response elements in the promoter regions of target genes. In humans, there are two main subtypes of estrogen receptors: estrogen receptor alpha (ESRα) and estrogen receptor beta (ESRβ), which are encoded by the *ESR1* and *ESR2* genes, respectively.

ESRα, which is expressed primarily in the ovary, uterus, and mammary glands, plays a key role in mediating the proliferative and developmental effects of estrogen, particularly in reproductive tissues^14^. In the ovary, ESRα is expressed in theca cells and supports the proliferative actions of estrogens in folliculogenesis^15^. ESRα is encoded by the *ESR1* gene, which is located on chromosome 6q25.1 and spans more than 140 kilobases. This gene comprises eight exons that contain several polymorphisms that can influence receptor expression and function. ESRβ is expressed in the ovary, especially in the granulosa cells of growing follicles, and helps in the differentiation of granulosa cells^14^. It is encoded by the *ESR2* gene located on chromosome 14q23.2 and is reported to modulate the effects of ESRα, often counteracting its proliferative actions and playing a significant role in follicular development and ovarian steroidogenesis^12^.

Several SNPs in the *ESR1* and *ESR2* genes have been studied in relation to ovarian response and reproductive outcomes^16,17^. The most studied polymorphisms of *ESR1* are c.453-397T > C (rs2234693) and c.453-351A > G (rs9340799), which have been linked to variations in ovarian reserve and response to controlled ovarian stimulation (COS)^18,19^. Reports also suggest that certain alleles of these SNPs are associated with a reduced ovarian response, lower antral follicle count (AFC) and low serum Anti-müllerian hormone (AMH)^20,21^. *ESR2* polymorphisms (rs1256049 and rs4986938) have also been implicated in ovarian function, with some studies reporting associations between specific *ESR2* genotypes and altered ovarian response. These SNPs may affect *ESR2* expression and its downstream effects on folliculogenesis and estradiol production^19^. These polymorphisms have also been associated with assisted reproduction outcomes^20^.

Over the years, several studies have explored the associations of *ESR* polymorphisms with the ovarian response to exogenous gonadotropin stimulation during ART cycles. Earlier systematic reviews and meta-analyses suggested a role of *ESR1* polymorphisms in diminished ovarian reserve and the risk of primary ovarian insufficiency^22,23^. As no meta-analysis has been conducted relating *ESR* gene polymorphisms with ovarian response, performing a meta-analysis to systematically assess the role of *ESR1*: c.453-397T > C (rs2234693) and c.453-351A > G (rs9340799); and *ESR2*: c.*39G > A (rs4986938) polymorphisms in the ovarian response is essential. The objective of this review was to identify the associations of *ESR1* and *ESR2* genetic polymorphisms with POR in ART treatment.

## Results

### Study identification and risk of bias analysis

**Fig. 1:**
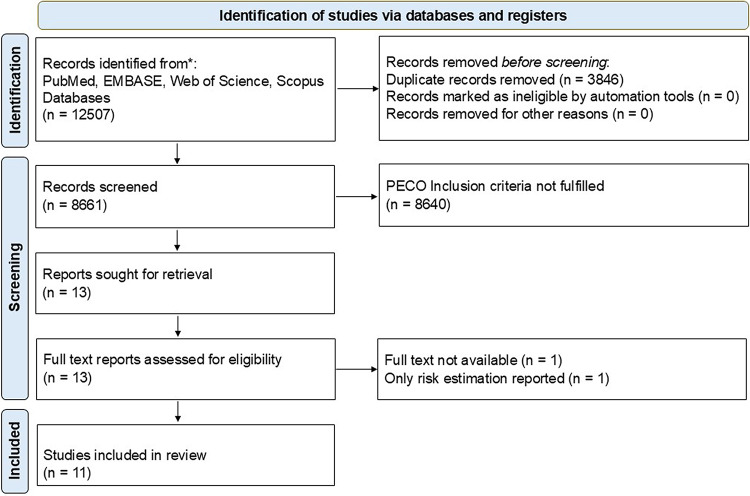
PRISMA flowchart of study selection. Flow chart of included and excluded studies.

**Fig. 2:**
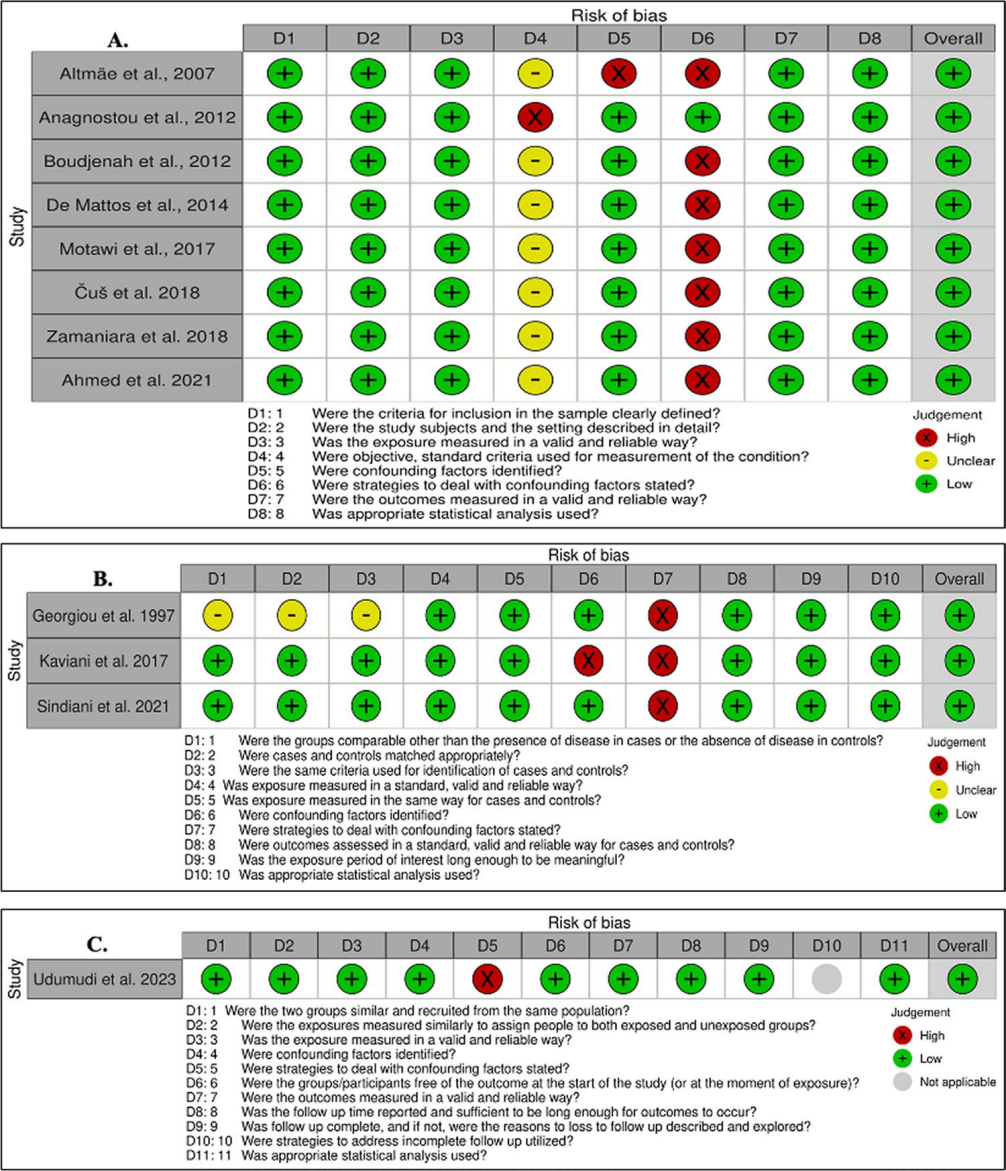
Representation of ROB analysis. Traffic light plots represent ROB analyzed using JBI critical appraisal tools for different types of study design: (**A**) Cross Sectional Study, (**B**) Case Control Study, (**C**) Cohort study.

A total of thirteen studies, which were matched with our PECO criteria, were included in this systematic review and meta-analysis. However, three studies were excluded for the meta-analysis because the full text was not available^24^, genotype details were not reported^25^, and risk of bias analysis demonstrated low quality of the study^30^ as reported in supplementary Table 1 (STable 1). A PRISMA flowchart of the study selection process is shown in Fig. 1. These studies were published between 2004 and 2023, and Table 1 presents the characteristics of the included studies. Among the ten included studies for meta-analysis, four originated from European countries^6,16,26,27^, whereas the remaining were reported from Iran^21,28^, Jordan^29^, Egypt^17,30^ and Brazil^20^. Among these studies, the sample size ranged from 17 to 428, with 1274 POR study subjects in total. Although several SNPs related to the *ESR* have been reported in relation to POR, this study considered two SNPs from *ESR1*: c.453–397 T > C (rs2234693), c.453-351A > G (rs9340799) and one SNP from *ESR*2: c.*39G > A (rs4986938) for meta-analysis. Allele frequency of *ESR1*: c.453–397 T > C (rs2234693) reported in GnomAD (https://gnomad.broadinstitute.org)^31^ was 0.4653, for *ESR1*: c.453-351A > G (rs9340799) was 0.3145 and for *ESR*2: c.*39G > A (rs4986938) was 0.3518^31^. All the studies followed Hardy Weinberg equilibrium (HWE) as provided in Table 1. Genotype frequency of SNPs included in meta-analysis is listed in supplementary table 2 (STable 2). Linkage disequilibrium was assessed for *ESR1*: c.453–397 T > C (rs2234693) and *ESR1*:c.453-351A > G (rs9340799) by D’ value (D' = 0.99360) and R^2^ value (R2 = 0.479). ROB was assessed using the checklists from JBI critical appraisal tools. Detailed assessment of ROB is shown in supplementary Table 3 (STable 3). Traffic light plots were created to visualize ROB analyzed using JBI critical appraisal tools for cross sectional studies (Fig. 2A), case control studies (Fig. 2B) and cohort studies (Fig. 2C). Based on the risk-of-bias assessment, one study^30^ was excluded due to its low methodological quality.

**Table 1:** Characteristics of included studies.

Sr. No	Author	Country	Study Design^#^	Total Sample size (n)	Sample size-POR (n)	Gene	SNP	Genotyping Method	POR Criteria^$^	Hardy–Weinberg Equilibrium followed
1	Georgiou et al.^30^	Greece	1, 2	100	100	*ESR1*	c.453-397T > C (rs2234693)	PCR–RFLP	–	Yes
2	Altmäe et al.^26^	Estonia	1, 3	149	149	*ESR1*	c.453-397T > C (rs2234693)	PCR–RFLP	1	Yes
							c.453-35A > G (rs9340799)			
						*ESR2*	c.984G > A (p.Val328Val, rs1256049)			
3	Anagnostou et al.^16^	Greece	1, 3	74	41	*ESR1*	c.453-397T > C (rs2234693)	PCR–RFLP	2	Yes
						*ESR2*	c.984G > A (p.Val328Val, rs1256049)			
4	Boudjenah et al.^27^	France	1, 3	428	428	*ESR1*	c.453-397T > C (rs2234693)	Sanger Sequencing	3	Yes
						*ESR2*	c.*39G > A (rs4986938)			
5	De Mattos et al.^20^	Brazil	1, 3	136	136	*ESR1*	c.453-397T > C (rs2234693)	TaqMan Allelic Discrimination Assay	1 or 3	Yes
							c.453-351A > G (rs9340799)			
						*ESR2*	c.*39G > A (rs4986938)			
							c.984G > A (p.Val328Val, rs1256049)			
6	Kaviani et al.^28^	Iran	1, 2	198	92	*ESR2*	c.*39G > A (rs4986938)	TaqMan Allelic Discrimination Assay	4	Yes
7	Motawi et al.^31^	Egypt	1, 3	216	105	*ESR2*	c.*39G > A (rs4986938)	TaqMan Allelic Discrimination Assay	3	Yes
8	Čuš et al.^6^	Slovenia	3	43	17	*ESR1*	c.453-397T > C (rs2234693)	High-Resolution Melting Analysis (HRMA)	1 and 5 or 6	Yes
9	Zamaniara et al.^21^	Iran	1, 3	186	94	*ESR1*	c.453-351A > G (rs9340799)	TaqMan Allelic Discrimination Assay	4	Yes
10	Ahmed et al.^17^	Egypt	3	172	92	*ESR1*	c.453-397T > C (rs2234693)	Sanger Sequencing	3	Yes
11	Sindiani et al.^29^	Jordan	1, 2	120	68	*ESR2*	c.984G > A (p.Val328Val, rs1256049)	PCR–RFLP	> 2 criteria: 4, 5, 6, 7	Yes
							c.*39G > A (rs4986938)			
12	Udumudi et al.^25^	India	1, 4	212	127	*ESR1*	c. 1782G > A (rs2228480), c. 229G > A (rs9340773), c. 261G > C (rs746432), c. 30T > C (rs2077647), c. 453-397T > C (rs2234693), c. 729T > C (rs4986934), c. 975G > C	Next-Generation Sequencing (NGS)	5 or 8 or 9	Yes
13	de Castro et al.^24^^***a***^	Spain	–	170	-	*ESR1*	–	–	1	Yes
						*ESR2*				

^a^Study characteristic retrieved from abstract, full text not available.

^#^Study design: 1. Observational, 2. Case- control, 3. Cross-sectional, 4. Cohort.

^$^POR Criteria: 1. ≤ 3 follicles/ oocytes, 2. serum E2 levels (< 40 mg/mL), 3. ≤ 4 mature oocytes, 4. ≤ 5 mature eggs/ oocytes, 5. AFC < 5 or < 9, 6. AMH < 1.1–0.5 ng/mL, 7. > 10 mIU/mL FSH on D3 of menstrual cycle, 8. total > 3000 units FSH consumption, 9. > 12 day stimulation,

### Association of *ESR1*: c.453-397 T > C (rs2234693) polymorphism with poor ovarian response

The connection between the *ESR1* gene (rs2234693) polymorphism and POR was studied through five studies^6,16,17,20,26^, including a sample size of 1009 (435 cases and 574 controls) for all the genetic models except for recessive model.

**Fig. 3:**
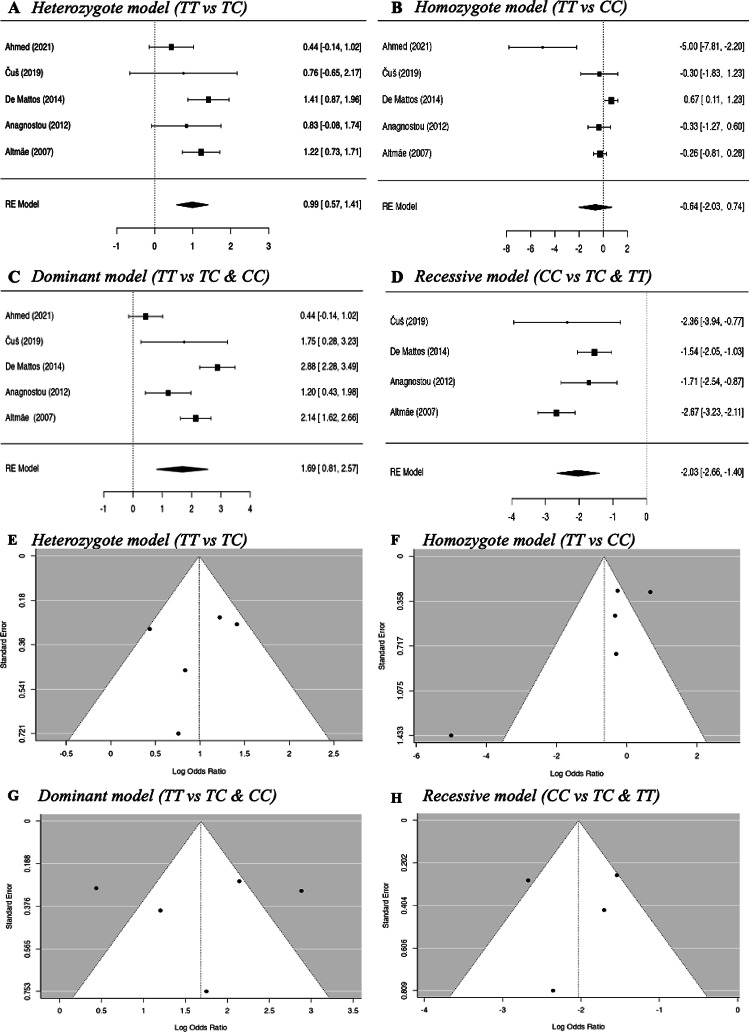
Meta-analysis of *ESR1* c.453-397 T > C (rs2234693) polymorphism. Forest plot representing the association of *ESR1* c.453-397 T > C (rs2234693) polymorphism with poor ovarian response, (**A**) heterozygote model, (**B**) homozygote model, (**C**) dominant model, (**D**) recessive model.

For the *ESR1* gene (rs2234693) polymorphism under the heterozygote model (TT vs. TC), heterozygotes with the TC genotype had higher odds of POR than those with the TT genotype (pooled log odds ratio, LOR = 0.99, standard error, SE = 0.213; odds ratio, OR = 2.69; Table 2, Fig. 3A). However, when the homozygote model (TT vs CC) was assessed, a weak and nonsignificant association was indicated between the CC genotype and POR compared with the TT genotype across the five studies (pooled LOR = -0.644, SE = 0.705, OR = 0.52; Table 2, Fig. 3B). When the dominant model (TT vs TC/CC) was considered, the pooled LOR was 1.69 (SE = 0.449), indicating that individuals carrying at least one C allele (TC or CC genotypes) had greater odds (OR = 5.41) of POR than those with the TT (wild type) genotype (Fig. 3C, Table 2). A leave-one-out sensitivity analysis was conducted on dominant model, exclusion of one study^20^ substantially reduced the pooled effect size (reduced from 2.17 to 0.53, which translates to an OR = 3.86), indicating that the inflated dominant-model estimate was largely driven by this single influential study. When the recessive model (TT/TC vs. CC) was considered, the pooled LOR was –3.29 (SE = 1.33), indicating that individuals with the CC genotype had significantly lower odds (OR = 0.03) of POR than those with the TT or TC genotypes (Fig. 3D, Table 2). A leave-one-out sensitivity analysis was conducted on recessive model, exclusion of^17^, which contained a zero TT genotype among cases, substantially reduced the pooled effect size (reduced from − 2.66 to − 1.4, which translates to an OR = 0.131), indicating that the extreme estimate was largely driven by sparse genotype counts.

**Table 2:** Random effect model estimates for the effect of *ESR* SNPs on poor ovarian response.

Gene	SNP	Genetic Model	Genotype	Estimate	SE	Z	p	CI	
								Lower Bound	Upper Bound
*ESR1*	c.453–397 T > C (rs2234693)	Heterozygote	TT vs TC	0.990	0.213	4.64	< 0.001	0.572	1.408
		Homozygote	TT vs CC	− 0.644	0.705	− 0.91	0.361	− 2.026	0.738
		Dominant	TT vs TC & CC	1.69	0.449	3.75	< 0.001	0.806	2.566
		Recessive	TT & TC vs CC	− 3.29	1.33	− 2.47	0.013	− 5.909	− 0.681
	Subgroup of c.453–397 T > C (rs2234693)	Heterozygote	TT vs TC	1.10	0.210	5.24	< 0.001	0.689	1.513
		Homozygote	TT vs CC	− 0.498	2.92	− 1.70	0.089	− 1.071	0.075
		Dominant	TT vs TC & CC	1.74	0.348	5.01	< 0.001	1.061	2.425
		Recessive	TT & TC vs CC	− 2.28	0.360	− 6.34	< 0.001	− 2.986	− 1.575
	c.453–351 A > G (rs9340799)	Heterozygote	AA vs AG	0.543	0.377	1.44	0.149	− 0.195	1.282
		Homozygote	AA vs GG	− 1.11	0.412	− 2.69	0.007	− 1.915	− 2.990
		Dominant	AA vs AG & GG	1.21	0.500	2.42	0.016	0.228	2.186
		Recessive	AA & AG vs GG	− 3.42	0.334	− 10.2	< 0.001	− 4.071	− 2.762
*ESR2*	c.*39 G > A (rs4986938)	Heterozygote	GG vs GA	0.568	0.202	2.81	0.005	0.172	0.963
		Homozygote	GG vs AA	− 0.968	0.399	− 2.43	0.015	− 1.750	− 0.186
		Dominant	GG vs GA & AA	1.29	0.365	3.52	< 0.001	0.570	2.003
		Recessive	GG & GA vs AA	− 3.23	0.447	− 7.22	< 0.001	− 4.106	− 2.352

### Association of *ESR1*: c.453-397 T > C (rs2234693) polymorphism with poor ovarian response in European population

Three studies^6,16,26^ featuring European population of 473 sample size (207 cases and 266 controls) were analyzed with sub-group meta-analysis for the association of *ESR1* gene (rs2234693) polymorphism with POR.

Heterozygote model (TT vs. TC) of the *ESR1* gene (rs2234693) polymorphism had higher risk of POR for the TC genotype (pooled LOR = 1.10, standard error, SE = 0.210; OR = 3.0; Table 2, SFig. 1A). Similarly, the dominant model (TT vs TC/CC) had higher odds of POR in subjects with TC/CC as significant and positive LOR was observed (pooled LOR = 1.74, standard error, SE = 0.348; OR = 5.69; Table 2, SFig. 1B). Conversely, under the homozygote (pooled LOR = − 0.498, standard error, SE = 2.92; OR = 0.60; Table 2, SFig. 1C) and recessive model (pooled LOR = − 2.28, standard error, SE = 0.360; OR = 0.10; Table 2, SFig. 1D), less risk of POR was observed in patients with TC and CC genotype. However, only significant LOR was observed in the recessive model and not in the homozygote model.

### Association of *ESR1*: c.453-351A > G (rs9340799) polymorphism with poor ovarian response

To identify the association between the *ESR1* (rs9340799) polymorphism and poor ovarian response, three studies were considered with a sample size of 714, 379 of which were cases and 335 of which were controls^20,21,26^.

**Fig. 4:**
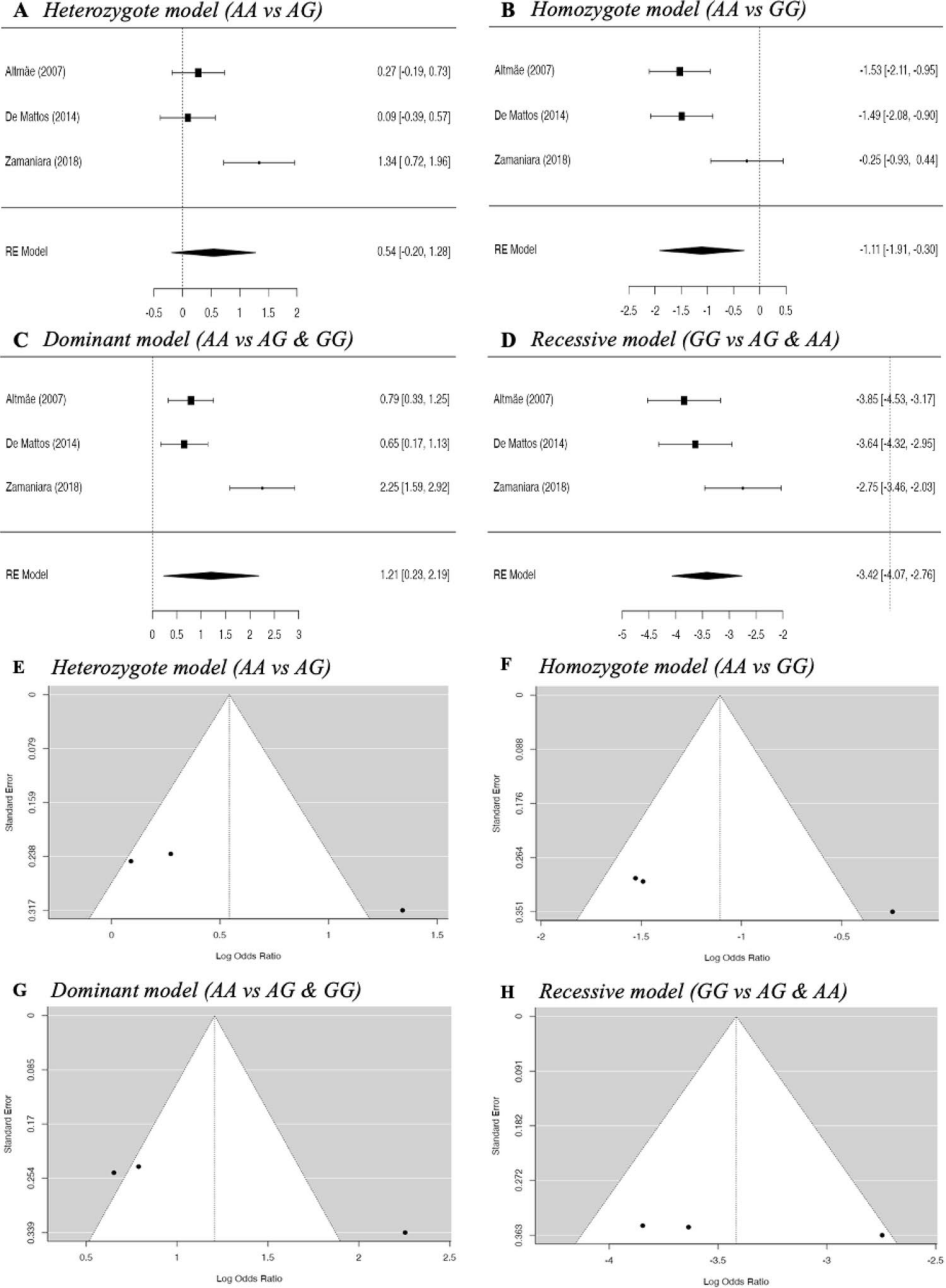
Meta-analysis of *ESR1* c.453-351A > G (rs9340799) polymorphism. Forest plot representing the association of *ESR1* c.453-351A > G (rs9340799) polymorphism with poor ovarian response, (**A**) heterozygote model, (**B**) homozygote model, (**C**) dominant model, (**D**) recessive model.

When the heterozygote model (AA vs. AG) was studied, the pooled LOR was 0.543 (SE = 0.377), indicating that individuals with the AG genotype had higher odds (OR = 1.72) of POR than those with the AA genotype (Fig. 4A, Table 2). In the homozygote model (AA vs. GG), the pooled LOR was –1.11 (SE = 0.412), indicating that individuals with the AA (wild-type) genotype had significantly lower odds (OR = 0.33) of POR than those with the GG genotype (Fig. 4B, Table 2). In the dominant model (AA vs. AG/GG), the pooled LOR was 1.21 (SE = 0.500, Table 2), indicating that individuals with the AG or GG genotype had higher odds (OR = 3.35) of POR than those with the AA (wild type) genotype (Fig. 4C,). When the recessive model (AA/AG vs. GG) was considered, the pooled LOR was –3.42 (SE = 0.334, Table 2), indicating that individuals with the GG genotype had significantly lower odds (OR = 0.03) of poor ovarian response (POR) than those with the AA or AG genotypes (Fig. 4D).

### Association of *ESR2*: c.*39G > A (rs4986938) polymorphism with poor ovarian response

To assess the association between the *ESR2* (rs4986938) polymorphism and POR, five studies^20,27–30^ with a total sample size of 1363 (829 cases, 534 controls) were included.

**Fig. 5:**
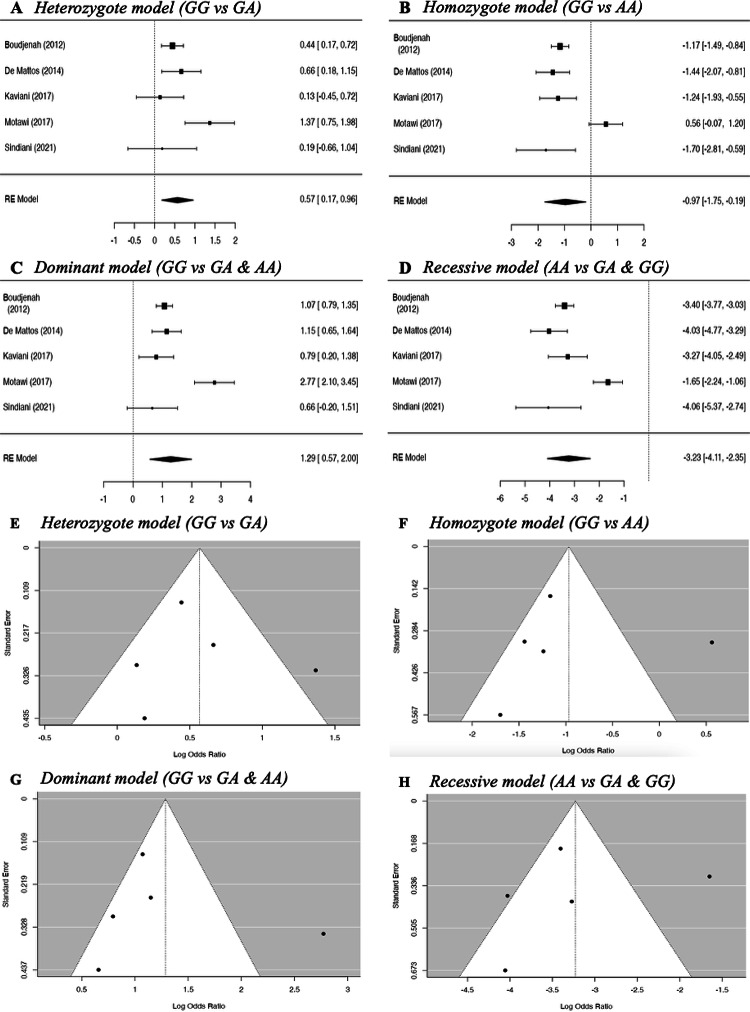
Meta-analysis of *ESR2* c.*39G > A (rs4986938) polymorphism. Forest plot representing the association of *ESR2* c.*39G > A (rs4986938) polymorphism with poor ovarian response, (**A**) heterozygote model, (**B**) homozygote model, (**C**) dominant model, (**D**) recessive model.

When the heterozygote model (GG vs GA) was considered, the pooled LOR was 0.568 (SE = 0.202), indicating that individuals with the GA genotype had higher odds (OR = 1.76) of POR than those with the GG (wild type) genotype (Fig. 5A, Table 2). These findings suggest a positive association between *ESR2* (rs4986938) and POR under the heterozygote model. When the homozygote model (GG vs AA) was considered, the pooled LOR was –0.968 (SE = 0.399), indicating that individuals with the AA genotype had lower odds (OR = 0.38) of POR than those with the GG (wild-type) genotype (Fig. 5B, Table 2). These findings suggest a negative association between *ESR2* (rs4986938) and POR under the homozygote model. When the dominant model (GG vs GA/AA) was considered, the pooled LOR was 1.29 (SE = 0.365), indicating that individuals with the GA or AA genotype had higher odds (OR = 3.63) of POR than those with the GG (wild type) genotype (Fig. 5C, Table 2). These findings suggest a positive association between *ESR2* (rs4986938) and POR under the dominant model. When the recessive model (GA/GG vs AA) was considered, the pooled LOR was –3.23 (SE = 0.447), indicating that individuals with the AA genotype had substantially lower odds (OR = 0.04) of POR than those with the GA or GG genotypes (Fig. 5D, Table 2). These findings suggest a strong negative association between *ESR2* (rs4986938) and POR under the recessive model.

### Sensitivity analysis

Meta-analysis of the *ESR1* gene (rs2234693) polymorphism and POR revealed low to moderate heterogeneity between the studies. The heterozygote model had modest between-study heterogeneity (I^2^ = 46.1%), whereas the other models had moderate to high heterogeneity between the studies (Table 3). Sub-group meta-analysis of *ESR1* gene (rs2234693) polymorphism had low heterogeneity in the heterozygote (I^2^ = 0%) and homozygote (I^2^ = 22.47%) model, while modest heterogeneity was observed in the dominant (I^2^ = 50.81%) and recessive (I^2^ = 47.31%, Table 3). In the *ESR1* (rs9340799) and POR analyses, one study out of three was identified as a potential outlier^21^ in all the study models analyzed (Table 4). No studies were classified as overly influential (Table 3). Between-study heterogeneity was substantial under all the models studied (I^2^ > 80%, Table 3), except for the recessive model, where it was moderate (I^2^ = 62.68%). In the case of the *ESR2* (rs4986938) polymorphism, one study out of three was identified as a potential outlier^30^, and no studies were considered overly influential (Table 3). The between-study heterogeneity was moderate (I^2^ = 65.69%, Table 3) for the heterozygote model and substantial for all the other models (I^2^ > 80%, Table 3).

**Table 3:** Sensitivity Analysis.

Gene	SNP	Genetic Model	Genotype	Tau	Tau^2^	SE	I^2^	H^2^	df	Q	p
*ESR1*	c.453–397 T > C (rs2234693)	Heterozygote	TT vs CT	0.31	0.099	0.15	46.18	1.858	4.0	6.81	0.146
		Homozygote	TT vs CC	1.42	2.034	1.744	91.5	11.759	4.0	19.28	< 0.001
		Dominant	TT vs TC & CC	0.91	0.84	0.710	87.47	7.981	4.0	37.24	< 0.001
		Recessive	CC vs TC & TT	2.84	8.099	6.269	97.86	47.765	4.0	26.84	< 0.001
	Subgroup of c.453–397 T > C (rs2234693)	Heterozygote	TT vs CT	0.0	0.0	0.1765	0.0	1.00	2.0	0.786	0.675
		Homozygote	TT vs CC	0.25	0.067	0.2868	22.47	1.290	2.0	2.188	0.335
		Dominant	TT vs TC & CC	0.42	0.182	0.3723	50.81	2.033	2.0	3.920	0.141
		Recessive	CC vs TC & TT	0.42	0.183	0.3995	47.31	1.898	2.0	3.564	0.168
	c.453–351 A > G (rs9340799)	Heterozygote	AA vs AG	0.59	0.354	0.426	83.74	6.151	2.0	10.612	0.005
		Homozygote	AA vs GG	0.64	0.409	0.509	80.39	5.101	2.0	9.502	0.009
		Dominant	AA vs AG & GG	0.82	0.672	0.749	90.28	10.290	2.0	16.421	< 0.001
		Recessive	GG vs AG & AA	0.45	0.209	0.334	62.68	2.680	2.0	5.326	0.070
*ESR2*	c.*39 G > A (rs4986938)	Heterozygote	GG vs GA	0.35	0.125	0.143	65.69	2.915	4.0	10.23	0.037
		Homozygote	GG vs AA	0.81	0.670	0.562	87.8	8.198	4.0	28.19	< 0.001
		Dominant	GG vs AG & AA	0.75	0.575	0.471	89.21	9.270	4.0	24.993	< 0.001
		Recessive	AA vs AG & GG	0.91	0.842	0.705	88.43	8.644	4.0	34.212	< 0.001

**Table 4:** Analysis of publication bias.

Gene	SNP	Genetic Model	Genotype	Kendall’s rank correlation		Egger’s regression	
				τ	p	z	p
*ESR1*	c.453–397 T > C (rs2234693)	Heterozygote	TT vs CT	− 0.200	0.817	–0.572	0.567
		Homozygote	TT vs CC	− 0.200	0.817	–2.913	0.004
		Dominant	TT vs TC & CC	0.000	1.00	− 0.064	0.949
		Recessive	CC vs TC & TT	− 0.600	0.233	− 3.366	< 0.001
	Subgroup of c.453–397 T > C (rs2234693)	Heterozygote	TT vs CT	− 0.333	1.0	–0.834	0.404
		Homozygote	TT vs CC	− 0.333	1.0	–0.279	0.780
		Dominant	TT vs TC & CC	− 0.333	1.0	− 0.317	0.752
		Recessive	CC vs TC & TT	0.333	1.0	0.242	0.809
	c.453–351 A > G (rs9340799)	Heterozygote	AA vs AG	0.333	1.0	3.092	0.002
		Homozygote	AA vs GG	1.0	0.333	3.075	0.002
		Dominant	AA vs AG & GG	0.333	1.0	3.967	< 0.001
		Recessive	GG vs AG & AA	1.0	0.333	2.306	0.021
*ESR2*	c.*39 G > A (rs4986938)	Heterozygote	GG vs GA	0.20	0.817	− 0.064	0.949
		Homozygote	GG vs AA	0.400	0.483	− 0.437	0.662
		Dominant	GG vs AG & AA	0.000	1.0	0.147	0.883
		Recessive	AA vs AG & GG	0.000	1.0	− 0.806	0.421

### Risk of publication bias

Egger’s regression test and funnel plots were used to identify bias and further strengthen the confidence level of the results. The funnel plot did not reveal evidence asymmetry for the *ESR1* (rs2234693) polymorphism under all the models considered (Fig. 3E-H), which indicated that there was no noticeable publication bias, and Egger’s test demonstrated that there was no statistical significance for the evaluation of publication bias under all the genetic models considered for the *ESR1* (rs2234693) polymorphism (Table 4). Similarly, in sub-group analysis of *ESR1* (rs2234693) polymorphism, no significant asymmetry and publication bias was observed as represented in supplementary figure (S1E-H Fig, Table 4). Furthermore, for the *ESR1* (rs9340799) polymorphism, the publication bias assessment revealed mixed results, with no asymmetry detected by Kendall’s rank correlation test (Table 4), but significant funnel plot asymmetry was observed via Egger’s regression test (Fig. 4E–H). No significant publication bias was evident for the *ESR2* (rs4986938) polymorphism (Fig. 5E–H; Table 4).

## Discussion

This study was conducted in an effort to elucidate the role of estrogen receptor SNPs in the ovarian response during COS. To the best of our knowledge, this is the first systematic review and meta-analysis to consider the role of *ESR1* and *ESR2* SNPs in the ovarian response of patients undergoing ovarian stimulation and ART treatment. Our findings indicate that women with certain *ESR* SNPs may have a greater risk of POR. In particular, C allele carriers of *ESR1* (rs2234693) may have an elevated risk of POR, although the observed low to moderate heterogeneity highlighted the need to analyze diverse populations. From the included studies, sub-group meta-analysis of the European population was assessed. Ethnic sub-group meta-analysis also revealed C allele carriers have a higher risk of POR, as observed from heterozygote and dominant models. Furthermore, G allele carriers of *ESR1* (rs9340799) and A allele carriers of *ESR2* (rs4986938) demonstrated increased susceptibility to POR. However, significant publication bias and heterogeneity in *ESR1* (rs9340799) and *ESR2* (rs4986938) polymorphisms respectively undermine the reliability of the findings. It has been observed that the studies included in this meta-analysis are almost clinically homogeneous in terms of classification of POR based on oocyte or follicle numbers and hence, the validity of the findings from the meta-analysis is not affected by the choice of studies available and included. However, these results should be interpreted with caution because of heterogeneity across studies, small sample sizes, population variability, and potential publication bias.

This study included ten studies with 1722 study subjects, 1274 of which were in the POR group. For various technical reasons, we could add five studies for *ESR1* (rs2234693) and three studies for *ESR1* (rs9340799) and five studies for *ESR2* (rs4986938) polymorphisms. The primary outcome of the study was POR post-COS, which was defined as < 3–5 oocytes in most of the studies. Although, there are no meta-analysis studies relating estrogen receptor SNPs with POR, studies have correlated these SNPs with premature ovarian insufficiency risk^22^.

Estrogen plays an important role in human reproduction and helps maintain the fertility potential of women. Polymorphisms associated with estrogen receptors have been associated with various gynecological conditions and pregnancy complications^12,32^. Previous meta-analyses have associated most studied *ESR1* and *ESR2* polymorphisms with poor ovarian insufficiency, preeclampsia and other gynecological conditions^33–35^.

Our literature search identified ten SNPs in the *ESR1* and *ESR2* genes associated with the ovarian response. The highly reported and studied SNPs for POR are c.453-397 T > C (rs2234693) and c.453-351A > G (rs9340799) in *ESR1* and *ESR2* c.*39G > A (rs4986938). Both rs2234693 and rs9340799 of the *ESR1* are in intron 1 of the gene. *ESR1* rs2234693 SNP is characterized by a T > C transition in intron 1, and rs2234693 has been reported to impact the binding of transcription factors, affecting alternative splicing, consequently, altering the gene expression and functionality^36–38^. As estrogen is involved in the active transcription of myb^39^, a change in nucleotides from T to C may cause decreased *ESR1* expression. This leads to impaired estrogen mediation by *ESR1*, resulting in a relative estrogen deficit. The *ESR1* rs9340799 polymorphism is reported to have significant functions; however, the exact mechanisms remain underexplored^40^. The *ESR2* gene, located on chromosome 14q22-24, expands into eight exons. *ESR2* rs4986938 is in the 3’ untranslated region (UTR) of the last exon^41^. *ESR2* rs4986938 is a noncoding change from a G to A nucleotide that is reported to be correlated with decreased expression of *ESR2*. *ESR2* rs4986938 is reported to play a crucial role in ovulatory dysfunctions, specifically in idiopathic cases^42^. Single nucleotide changes in *ESR2* rs4986938 alter mRNA stability and translatability^33,43^.

A study by Asgari et al.^44^ reported that the *ESR1* rs2234693 and rs9340799 polymorphic genotypes were associated with a significantly lower number of good-quality embryos^45^. *ESR1* rs2234693 affects various aspects of fertility, including oocyte number, AFC, maturation and the pregnancy rate^44^. *ESR1* rs9340799 has been reported to be associated with a decrease in the number of follicles, oocytes and implantation failure^46,47^. Compared with the wild-type and heterozygote genotypes, *ESR2* rs49866938 was correlated with a reduced oocyte number, maturity and, hence, possibly, a poor response^27,48^. Consistent with the findings of the literature, the meta-analysis revealed an association of *ESR1* rs2234693 with a high risk of POR in patients with the TC and CC genotypes, whereas patients with the polymorphic genotype of *ESR1* rs9340799 had significantly lower odds of POR; however, substantial heterogeneity among studies was observed. Patients with *ESR2* rs4986938 GA & AA genotypes under the dominant model are likely to present POR compared with other genetic models.

We acknowledge that the current meta-analysis has several limitations. First, the studies included in this meta-analysis were observational and retrospective in nature. Thus, it is more susceptible to bias. Further, the available reports for each SNP were limited, with a small number of studies contributing to the meta‑analysis, which precluded meaningful stratified analyses by study design (case‑control, cross‑sectional, or cohort). To minimize potential bias arising from differing study designs, random‑effects models were applied throughout the analyses. Furthermore, there were considerable variations across studies in how data were reported, indicating that studies correlating COS outcomes with patient genotype are limited. Hence, the study could not consider characteristics such as FSH consumption during COS, type of COS protocol, duration of COS, number of mature oocytes, fertilization and implantation status as secondary outcomes to explore their association with a specific SNP analyzed. In addition, considerable variation was observed in terms of the age of the study subjects, and the criteria used to define POR and the method used for genotyping.

Secondly, the leave-one-out sensitivity analysis was performed only for *ESR1*: c.453-397 T > C (rs2234693). However, it was not performed for other SNPs as there were limited studies and outliers were detected, which might have influenced the study results. Third, when individual SNPs are considered, the sample size is small. To address this limitation, in addition random‑effects models, sensitivity analyses was performed where feasible to assess the stability of the pooled estimates. For *ESR1* rs2234693, sensitivity analysis did not identify significant outliers or evidence of publication bias. For *ESR1* rs9340799 and *ESR2* rs4986938, a single influential study was detected; however, due to the limited number of available studies, further stratified or power‑adjusted analyses were not feasible. Hence, the results should be interpreted cautiously.

Fourth, the studies included in our review were heterogeneous. The substantial heterogeneity is likely attributable to differences in study populations (ethnicity), small and uneven sample sizes, variations in ovarian stimulation protocols, and heterogeneity in the definition of poor ovarian response across included studies. To account for this variability, random‑effects models and sensitivity analyses, including leave‑one‑out analysis where feasible, were applied to evaluate the influence of individual studies on pooled estimates. However, further exploration of heterogeneity through meta‑regression was not performed, as the number of studies per SNP was fewer than ten in most analyses, consistent with Cochrane Handbook recommendations.

Owing to the limited number of studies representing diverse populations for specific SNPs, only one sub-group meta-analysis for *ESR1* rs2234693 was performed. While meta-analysis of genetic models with *ESR1* rs9340799 show lower odds of POR with wild-type genotype, the observations may not apply to all ethnicities as only three studies were analyzed for rs9340799, that represents niche ethnic populations and demonstrated significant publication bias. Further, sub-group analysis within populations could not be performed due to limited studies.

Additionally, articles/reports published in English language were included, this could lead to exclusion of relevant studies published in other languages, leading to language bias. Furthermore, our sensitivity analysis revealed that the studies included for *ESR1* rs2234693 did not show any outliers or publication bias. Although funnel plots and Egger’s regression test were generated, their interpretability is limited because fewer than 10 studies were available for each genetic model. Both visual and statistical assessments of publication bias are known to be unreliable and underpowered with small study numbers: therefore, the results should be interpreted cautiously, and the possibility of publication bias cannot be ruled out. The other SNPs considered had one study each that were outliers. However, pooled effect sizes and low to modest heterogeneity indicated a correlation of heterozygote models of *ESR1* rs2234693 and *ESR2* rs4986938 with POR. Hence, the findings of this study are reliable.

As the current literature consists only of observational and retrospective studies on this topic, trials should be conducted with larger sample sizes and better study designs. Further, most of the studies have considered single genes while correlating with COS outcomes. Future studies should include multiple relevant genes to better understand the polygenic traits of POR. Furthermore, most of the studies consider patients with < 5 oocytes to be poor responders. Importantly, there are young patients with suboptimal responses, which may need specific attention. Hence, future studies should consider this subgroup to identify the genomic risk with a large cohort of patients. Moreover, pharmacogenomic approaches are limited in this field. These approaches are needed in the future and may help to better understand POR pathophysiology and improve ART outcomes. Accordingly, robust, large‑scale studies across diverse populations integrating genetic, clinical, and treatment data are needed to clarify the role of *ESR* polymorphisms in personalized ART.

## Conclusion

In conclusion, this meta-analysis suggests that the *ESR1* rs2234693 polymorphism may be associated with an increased risk of poor ovarian response in women undergoing controlled ovarian stimulation, particularly in European populations. However, the current evidence is limited by heterogeneity across studies, small sample sizes, population variability and publication bias, all of which warrant cautious interpretation. Importantly, these findings are not yet sufficient to support any changes to ART protocols or clinical decision making. Consequently, there is a need for large-scale, well-designed, and ethnically diverse studies integrating genetic data with detailed clinical and treatment parameters to clarify the potential role of *ESR* polymorphisms in personalized ART strategies.

## Methods

### Protocol and registration

This review has adhered to the Preferred Reporting Items for Systematic Reviews and Meta-analysis (PRISMA) guidelines for systematic reviews and meta-analyses^49^ and provides a checklist in the supplementary table 1 (S.Table 1). The study protocol was registered at http://www.crd.york.ac.uk/PROSPERO/(CRD42024572991) on August 2, 2024.

### Eligibility criteria

The study followed the PECO (Patients, Exposure, Comparator and Outcomes) model to select the study population. The PECO goal was to identify the associations of *ESR1* and *ESR2* genetic polymorphisms with POR in ART treatment. No time limits were applied, and queries were limited to human studies.

### Inclusion criteria

All types of studies with patients undergoing COS and classified as POR after stimulation were included. Age of patients ranged between 20 to 46 years old. Normal ovarian response (NOR) and POR classified patients were genotyped for various *ESR1* and *ESR2* gene polymorphisms. Control patients (NOR) were defined by: normally ovulating infertile/fertile women, patients with normal ovarian reserve, normal ovulatory cycles, normal hormone levels, oocytes retrieved > 5 to < 15. POR cases were defined by one or combination of following criteria: number of oocytes retrieved (≤ 3 to ≤ 5), mature oocytes(≤ 4), follicles/ growing follicles (≤ 3), mature eggs (≤ 5) or ovarian follicles (< 3), AFC (< 9 or < 5), hormone levels of AMH (< 1.1 ng/mL or < 0.5 ng/mL), serum E2(< 40 mg/mL), FSH (< 40 mg/mL) or total FSH consumption (> 3000 units).

### Exclusion criteria

Studies that were excluded are as follows: (i) studies which did not meet the PECO criteria where patients with any other pathological condition affecting ovarian response were considered; (ii) studies with incomplete or insufficient genotype data^25^; (iii) studies where full text not available^24^; (iv) editorials, reviews, letters, commentaries, meta-analysis (v) duplicate records.

### Search strategy

A systematic search of PubMed, EMBASE, SCOPUS and Web of Science databases was conducted to identify all relevant studies published up to October 9, 2025. Combinations of the following keywords, Boolean operators and database specific controlled vocabulary were used: POR, poor responders*, poor ovarian response, controlled ovarian stimulation, in vitro fertilization, assisted reproductive technology, estrogen receptor*, estrogen*, ESR*. Detailed search strategy has been provided in supplementary table 5 (S Table 5). The search was restricted to English‑language publications from database inception until October 9, 2025. Manual searches and reference checking of included studies and relevant reviews were performed to identify additional eligible studies^27^. The present review did not include grey literature and conference proceedings.

The search strategy was developed in consultation with an experienced subject experts both in evidence synthesis and reproductive genetics.

### Selection of studies

Following database searches, all retrieved records were imported into Rayyan (https://www.rayyan.ai/cite/) software^52^ and duplicate citations were identified and removed prior to screening. Study selection was performed independently by two reviewers (KZF and SI) through title/abstract and full‑text screening. Any discrepancies between reviewers were resolved through discussion, and when consensus could not be reached, a senior author was consulted for final decision‑making (SU)^50^.

### Data extraction

The data were extracted independently by two reviewers (KZF and SI) via predefined data fields, and the study quality indicators were entered into an Excel spreadsheet which was pilot tested. Discrepancies were resolved by discussion. The following items were extracted from each eligible study: the authors, year of publication, country, sample size, gene, and SNP-described genotype frequency/distribution. Data were extracted for all *ESR1* and *ESR2* SNPs present in the included studies. For full text articles not available, authors were contacted via email where available or a request for the article was made via the institutional library.

### Risk of bias analysis

Risk of bias (ROB) was evaluated with case–control, cross-sectional and cohort studies checklist by JBI (The Joanna Briggs Institute) critical appraisal tools^51^. Each study was assessed by two reviewers independently (KZF and VLR).

### Statistical analysis

Statistical analysis was carried out via Jamovi v2.3.28 (https://www.jamovi.org/about.html)^52^. Categorical data were combined with a pooled odds ratio (OR) via the Mantel–Haenszel method. Continuous data were combined with weighted mean differences (WMDs) via the inverse variance method. When at least three studies were available, a meta-analysis was conducted via the random effects (RE) model. RE model was applied to account for the heterogeneity in POR criteria, COS methods, patient age and ethnicity. Meta-analysis was performed for four genetic models: heterozygote, homozygote, dominant and recessive. Sub-group meta-analysis was performed on the stratified data (by region) for SNPs whenever there were adequate (3 or more) studies to carry out the same. Heterogeneity was assessed via the percentage of total variation in the estimated effect across studies via a chi-square-based Q test^53^ combined with the inconsistency index (I^2^) and DerSimonian‒Laird estimator^54^. An I^2^ value > 50% indicates substantial heterogeneity. The results were considered statistically significant when the p value was < 0.05. We applied Bonferroni correction in cases of statistical significance. The strength of the association between the SNPs and ovarian response was evaluated based on ORs and 95% confidence intervals (CIs). Linkage disequilibrium was calculated by LDpair Tool (https://ldlink.nih.gov)^55^.

### Publication bias

The quality of the studies included in this meta-analysis were evaluated by two authors (KZF and VLR), who assessed the publication bias of each study by Jamovi v2.3.28^56^. Egger’s regression and Kendall’s rank correlation was used to evaluate the publication bias in the studies included. The analysis included estimating the publication bias for reporting the group size and genotype frequency of the SNP. Bias across studies regarding the primary outcome was assessed via visual inspection of funnel plots^57^.

## Supplementary Information

Below is the link to the electronic supplementary material.

Supplementary Material 1

## Data Availability

The datasets used and/or analyzed during the current study are available from the corresponding author upon reasonable request.
