# Supplementary material for: Effect of ESR1 and ESR2 polymorphisms on ovarian response in assisted reproductive technology cycles: a systematic review and meta-analysis
Source: Sci Rep. 2026 Jun 5;16:25749. doi: 10.1038/s41598-026-53350-5 (PMC13482574; doi:10.1038/s41598-026-53350-5)
Supplement: Supplementary file 1 [file 41598_2026_53350_MOESM1_ESM.docx]

**Supplementary Information**

**S. Table 1 Excluded articles not included for meta-analysis, after full text screening and justification**

| **Study details** | **Justification** |
| --- | --- |
| De Castro F, Morón FJ, Montoro L, Galán JJ, Pérez- Hernández D, Sánchez-Casas Padilla E, et al. Human controlled ovarian hyperstimulation outcome is a polygenic trait. Pharmacogenetics. 2004 May;14(5):285–93. Available from: <https://pubmed.ncbi.nlm.nih.gov/15115914/>. Last accessed on 9^th^ October, 2024. | Full text not available |
| Udumudi A, Chaitra Lava K, Hegde V. High-Risk Genotypes Associated with Poor Response to Controlled Ovarian Stimulation in Indian Women. J Hum Reprod Sci. 2023 Jan 1;16(1):70. Available from: <https://pmc.ncbi.nlm.nih.gov/articles/PMC10256945/>. Last accessed on 9^th^ October, 2024. | Genotype details not reported, only risk estimation reported |
| Georgiou I, Konstantelli M, Syrrou M, Messinis IE, Lolis DE. Oestrogen receptor gene polymorphisms and ovarian stimulation for in-vitro fertilization A number of ESR gene polymorphisms and mutations have. Hum Reprod 1997;12(7):1430–3. | Excluded due to low study quality based on risk‑of‑bias assessment. |

**S Table 2: Genotype data from the included studies**

| SNP | Study | Wild type genotype | Polymorphic heterozygous genotype | Polymorphic homozygous genotype |
| --- | --- | --- | --- | --- |
| *ESR1*: c.453-397T>C (rs2234693) | Altmäe et al. 2007 (26) | 38 | 80 | 31 |
|  | Anagnostou et al. 2012 (16) | 12 | 20 | 9 |
|  | De Mattos et al. 2014 (20) | 26 | 67 | 43 |
|  | Čuš et al. 2018 (6) | 5 | 8 | 4 |
|  | Ahmed et al. 2021 (17) | 41 | 51 | 0 |
| *ESR1*: c.453-35A>G (rs9340799) | Altmäe et al. 2007 (26) | 60 | 70 | 19 |
|  | De Mattos et al. 2014 (20) | 57 | 60 | 19 |
|  | Zamaniara et al. 2018 (21) | 23 | 52 | 19 |
| *ESR2*: c.*39G>A (rs4986938) | Boudjenah et al. 2012 (27) | 158 | 204 | 66 |
|  | De Mattos et al. 2014 (20) | 49 | 71 | 16 |
|  | Kaviani et al. 2017 (28) | 37 | 40 | 15 |
|  | Motawi et al. 2017 (31) | 21 | 52 | 32 |
|  | Sindiani et al. 2021 (29) | 18 | 20 | 5 |

**S Table 3. ROB assessment from JBI critical appraisal tools checklists**

| **Cross Sectional Study** | | | | | | | | | |
| --- | --- | --- | --- | --- | --- | --- | --- | --- | --- |
| **Study ID** | 1. | 2. | 3. | 4. | 5. | 6. | 7. | 8. | Overall |
| Altmäe et al., 2007 | Low | Low | Low | Some concerns | High | High | Low | Low | Low |
| Anagnostou et al., 2012 | Low | Low | Low | High | Low | High | Low | Low | Low |
| Boudjenah et al., 2012 | Low | Low | Low | Some concerns | Low | High | Low | Low | Low |
| De Mattos et al., 2014 | Low | Low | Low | Some concerns | Low | High | Low | Low | Low |
| Motawi et al., 2017 | Low | Low | Low | Some concerns | Low | High | Low | Low | Low |
| Čuš et al. 2018 | Low | Low | Low | Some concerns | Low | High | Low | Low | Low |
| Zamaniara et al. 2018 | Low | Low | Low | Some concerns | Low | High | Low | Low | Low |
| Ahmed et al. 2021 | Low | Low | Low | Some concerns | Low | High | Low | Low | Low |

1. Were the criteria for inclusion in the sample clearly defined?
2. Were the study subjects and the setting described in detail?
3. Was the exposure measured in a valid and reliable way?
4. Were objective, standard criteria used for measurement of the condition?
5. Were confounding factors identified?
6. Were strategies to deal with confounding factors stated?
7. Were the outcomes measured in a valid and reliable way?
8. Was appropriate statistical analysis used?

| **Case Control Study** | | | | | | | | | | | | | |
| --- | --- | --- | --- | --- | --- | --- | --- | --- | --- | --- | --- | --- | --- |
| Study ID | 1. | 2. | | 3. | | 4. | 5. | 6. | 7. | 8. | 9. | 10. | Overall |
| Georgiou et al. 1997 | Some concerns | | Some concerns | | Some concerns | Low | Low | Low | High | Low | Low | Low | Low |
| Kaviani et al. 2017 | Low | Low | | Low | | Low | Low | High | High | Low | Low | Low | Low |
| Sindiani et al. 2021 | Low | Low | | Low | | Low | Low | Low | High | Low | Low | Low | Low |

1. Were the groups comparable other than the presence of disease in cases or the absence of disease in controls?
2. Were cases and controls matched appropriately?
3. Were the same criteria used for identification of cases and controls?
4. Was exposure measured in a standard, valid and reliable way?
5. Was exposure measured in the same way for cases and controls?
6. Were confounding factors identified?
7. Were strategies to deal with confounding factors stated?
8. Were outcomes assessed in a standard, valid and reliable way for cases and controls?
9. Was the exposure period of interest long enough to be meaningful?
10. Was appropriate statistical analysis used?

| **Cohort Study** | | | | | | | | | | | |
| --- | --- | --- | --- | --- | --- | --- | --- | --- | --- | --- | --- |
| Study ID | 1. | 2. | 3. | 4. | 5. | 6. | 7. | 8. | 9. | 10. | 11. |
| Udumudi et al. 2023 | Low | Low | Low | Low | High | Low | Low | Low | Low | Not applicable | Low |

1. Were the two groups similar and recruited from the same population?
2. Were the exposures measured similarly to assign people to both exposed and unexposed groups?
3. Was the exposure measured in a valid and reliable way?
4. Were confounding factors identified?
5. Were strategies to deal with confounding factors stated?
6. Were the groups/participants free of the outcome at the start of the study (or at the moment of exposure)?
7. Were the outcomes measured in a valid and reliable way?
8. Was the follow up time reported and sufficient to be long enough for outcomes to occur?
9. Was follow up complete, and if not, were the reasons to loss to follow up described and explored?
10. Were strategies to address incomplete follow up utilized?
11. Was appropriate statistical analysis used?

**S Table 4. PRISMA 2020 Checklist**

| **Section and Topic** | **Item #** | **Checklist item** | **Reported on Page** |
| --- | --- | --- | --- |
| **TITLE** | | |  |
| Title | 1 | Identify the report as a systematic review. | 1 |
| **ABSTRACT** | | |  |
| Abstract | 2 | Provided structured summary of objective, data source, synthesis methods, results, conclusion | 2 |
| **INTRODUCTION** | | |  |
| Rationale | 3 | Describe the rationale for the review in the context of existing knowledge. | 3 |
| Objectives | 4 | Provide an explicit statement of the objective(s) or question(s) the review addresses. | 5 |
| **METHODS** | | |  |
| Eligibility criteria | 5 | Specify the inclusion and exclusion criteria for the review and how studies were grouped for the syntheses. | 15-16 |
| Information sources | 6 | Specify all databases, registers, websites, organisations, reference lists and other sources searched or consulted to identify studies. Specify the date when each source was last searched or consulted. | 17 |
| Search strategy | 7 | Present the full search strategies for all databases, registers and websites, including any filters and limits used. | 17 |
| Selection process | 8 | Specify the methods used to decide whether a study met the inclusion criteria of the review, including how many reviewers screened each record and each report retrieved, whether they worked independently, and if applicable, details of automation tools used in the process. | 17 |
| Data collection process | 9 | Specify the methods used to collect data from reports, including how many reviewers collected data from each report, whether they worked independently, any processes for obtaining or confirming data from study investigators, and if applicable, details of automation tools used in the process. | 17-18 |
| Data items | 10a | List and define all outcomes for which data were sought. Specify whether all results that were compatible with each outcome domain in each study were sought (e.g. for all measures, time points, analyses), and if not, the methods used to decide which results to collect. | 17-18 |
|  | 10b | List and define all other variables for which data were sought (e.g. participant and intervention characteristics, funding sources). Describe any assumptions made about any missing or unclear information. | 17-18 |
| Study risk of bias assessment | 11 | Specify the methods used to assess risk of bias in the included studies, including details of the tool(s) used, how many reviewers assessed each study and whether they worked independently, and if applicable, details of automation tools used in the process. | 18 |
| Effect measures | 12 | Specify for each outcome the effect measure(s) (e.g. risk ratio, mean difference) used in the synthesis or presentation of results. | 18 |
| Synthesis methods | 13a | Describe the processes used to decide which studies were eligible for each synthesis (e.g. tabulating the study intervention characteristics and comparing against the planned groups for each synthesis (item #5)). | 18 |
|  | 13b | Describe any methods required to prepare the data for presentation or synthesis, such as handling of missing summary statistics, or data conversions. | - |
|  | 13c | Describe any methods used to tabulate or visually display results of individual studies and syntheses. | 18 |
|  | 13d | Describe any methods used to synthesize results and provide a rationale for the choice(s). If meta-analysis was performed, describe the model(s), method(s) to identify the presence and extent of statistical heterogeneity, and software package(s) used. | 18 |
|  | 13e | Describe any methods used to explore possible causes of heterogeneity among study results (e.g. subgroup analysis, meta-regression). | 18 |
|  | 13f | Describe any sensitivity analyses conducted to assess robustness of the synthesized results. | 18 |
| Reporting bias assessment | 14 | Describe any methods used to assess risk of bias due to missing results in a synthesis (arising from reporting biases). | 19 |
| Certainty assessment | 15 | Describe any methods used to assess certainty (or confidence) in the body of evidence for an outcome. | 18-19 |
| **RESULTS** | | |  |
| Study selection | 16a | Describe the results of the search and selection process, from the number of records identified in the search to the number of studies included in the review, ideally using a flow diagram. | 5-6 |
|  | 16b | Cite studies that might appear to meet the inclusion criteria, but which were excluded, and explain why they were excluded. | 5-6 |
| Study characteristics | 17 | Cite each included study and present its characteristics. | 5-6 |
| Risk of bias in studies | 18 | Present assessments of risk of bias for each included study. | 6 |
| Results of individual studies | 19 | For all outcomes, present, for each study: (a) summary statistics for each group (where appropriate) and (b) an effect estimate and its precision (e.g. confidence/credible interval), ideally using structured tables or plots. | 6-9 |
| Results of syntheses | 20a | For each synthesis, briefly summarise the characteristics and risk of bias among contributing studies. | 6-9 |
|  | 20b | Present results of all statistical syntheses conducted. If meta-analysis was done, present for each the summary estimate and its precision (e.g. confidence/credible interval) and measures of statistical heterogeneity. If comparing groups, describe the direction of the effect. | 6-9 |
|  | 20c | Present results of all investigations of possible causes of heterogeneity among study results. | 13-14 |
|  | 20d | Present results of all sensitivity analyses conducted to assess the robustness of the synthesized results. | 9- 10 |
| Reporting biases | 21 | Present assessments of risk of bias due to missing results (arising from reporting biases) for each synthesis assessed. | - |
| Certainty of evidence | 22 | Present assessments of certainty (or confidence) in the body of evidence for each outcome assessed. | 6-9 |
| **DISCUSSION** | | |  |
| Discussion | 23a | Provide a general interpretation of the results in the context of other evidence. | 11 |
|  | 23b | Discuss any limitations of the evidence included in the review. | 13-14 |
|  | 23c | Discuss any limitations of the review processes used. | 13-14 |
|  | 23d | Discuss implications of the results for practice, policy, and future research. | 14 |
| **OTHER INFORMATION** | | |  |
| Registration and protocol | 24a | Provide registration information for the review, including register name and registration number, or state that the review was not registered. | 15 |
|  | 24b | Indicate where the review protocol can be accessed, or state that a protocol was not prepared. | 15 |
|  | 24c | Describe and explain any amendments to information provided at registration or in the protocol. | - |
| Support | 25 | Describe sources of financial or non-financial support for the review, and the role of the funders or sponsors in the review. | 29 |
| Competing interests | 26 | Declare any competing interests of review authors. | 29 |
| Availability of data, code and other materials | 27 | Report which of the following are publicly available and where they can be found: template data collection forms; data extracted from included studies; data used for all analyses; analytic code; any other materials used in the review. | 29 |

**S Table 5: Search Strategy used in this study**

| Database | Search Strategy |
| --- | --- |
| PubMed | (("POR"[Title/Abstract] OR "poor responders*"[Title/Abstract] OR "poor ovarian response"[Title/Abstract] OR "controlled ovarian stimulation"[Title/Abstract] OR "COS"[Title/Abstract] OR "ovarian stimulation*"[Title/Abstract] OR "invitro fertilization*"[Title/Abstract] OR "ivf"[Title/Abstract] OR "assisted reproductive technology*"[Title/Abstract] OR "ART"[Title/Abstract]) AND ("estrogen receptor*"[Title/Abstract] OR "estrogen*"[Title/Abstract] OR "esr"[Title/Abstract])) AND ((casereports[Filter] OR classicalarticle[Filter] OR clinicalstudy[Filter] OR clinicaltrial[Filter] OR clinicaltrialphasei[Filter] OR clinicaltrialphaseii[Filter] OR clinicaltrialphaseiii[Filter] OR clinicaltrialphaseiv[Filter] OR comparativestudy[Filter] OR controlledclinicaltrial[Filter] OR multicenterstudy[Filter] OR observationalstudy[Filter] OR randomizedcontrolledtrial[Filter] OR twinstudy[Filter]) AND (humans[Filter]) AND (female[Filter]) AND (english[Filter])) |
| Scopus | ( estrogen AND receptor* OR estrogen* OR esr ) AND ( ( TITLE-ABS-KEY ( por ) OR TITLE-ABS-KEY ( poor AND responders* ) OR TITLE-ABS-KEY ( poor AND ovarian AND response ) OR TITLE-ABS-KEY ( controlled AND ovarian AND stimulation ) OR TITLE-ABS-KEY ( cos ) OR TITLE-ABS-KEY ( ovarian AND stimulation* ) OR TITLE-ABS-KEY ( invitro AND fertilization* ) OR TITLE-ABS-KEY ( ivf ) OR TITLE-ABS-KEY ( assisted AND reproductive AND technology* ) OR TITLE-ABS-KEY ( art ) ) ) |
| EMBASE | por OR 'poor responders':ab,ti OR 'poor ovarian response':ab,ti OR 'controlled ovarian stimulation':ab,ti OR cos:ab,ti OR 'ovarian stimulation':ab,ti OR 'invitro fertilization':ab,ti OR ivf:ab,ti OR 'assisted reproductive technology':ab,ti AND 'estrogen receptor' OR 'estrogen':ab,ti OR esr:ab,ti AND ('case control study'/de OR 'case report'/de OR 'case study'/de OR 'clinical article'/de OR 'clinical trial'/de OR 'comparative study'/de OR 'controlled clinical trial'/de OR 'human'/de OR 'multicenter study'/de OR 'observational study'/de OR 'phase 1 clinical trial'/de OR 'phase 2 clinical trial'/de OR 'phase 3 clinical trial'/de OR 'prospective study'/de OR 'randomized controlled trial'/de) AND 'Article'/it |
| Web of Science | POR (Abstract) or Poor Responders* (Abstract) or Poor Ovarian Response (Abstract) or Controlled Ovarian Stimulation (Abstract) or COS (Abstract) or Ovarian Stimulation (Abstract) or Invitro Fertilization (Abstract) or IVF (All Fields) or Assisted Reproductive Technology (All Fields) or ART (All Fields) AND estrogen receptor* (Abstract) or estrogen* (Abstract) or ESR (Abstract) |

**S Figure 1.** Forrest plot representing the association in subgroup of *ESR1* with c.453-397T>C (rs2234693) polymorphism with poor ovarian response, A) heterozygote model, B) homozygote model, C) dominant model, D) recessive model; funnel plot depicting the outliers of the *ESR1* c.453-397T>C (rs2234693) meta-analysis: E) heterozygote model, F) homozygote model, G) dominant model, H) recessive model.
